# The Cellular DExD/H-Box RNA Helicase UAP56 Co-localizes With the Influenza A Virus NS1 Protein

**DOI:** 10.3389/fmicb.2018.02192

**Published:** 2018-09-12

**Authors:** Shiho Chiba, Lindsay Hill-Batorski, Gabriele Neumann, Yoshihiro Kawaoka

**Affiliations:** ^1^Influenza Research Institute, Department of Pathobiological Sciences, School of Veterinary Medicine, University of Wisconsin-Madison, Madison, WI, United States; ^2^Division of Virology, Department of Microbiology and Immunology, Institute of Medical Science, University of Tokyo, Tokyo, Japan; ^3^International Research Center for Infectious Diseases, Institute of Medical Science, University of Tokyo, Tokyo, Japan

**Keywords:** influenza A virus, UAP56, influenza A NS1, nuclear localization, host factors

## Abstract

UAP56, a member of the DExD/H-box RNA helicase family, is essential for pre-mRNA splicing and mRNA export in eukaryotic cells. In influenza A virus-infected cells, UAP56 mediates viral mRNA nuclear export, facilitates viral ribonucleoprotein complex formation through direct interaction with the viral nucleoprotein, and may indirectly affect antiviral host responses by binding to and/or facilitating the activation of the antiviral host factors MxA and PKR. Here, we demonstrate that UAP56 also co-localizes with the influenza A viral NS1 protein, which counteracts host cell innate immune responses stimulated by virus infection. The UAP56–NS1 association relies on the RNA-binding residues R38 and K41 in NS1 and may be mediated by single-stranded RNA. UAP56 association with NS1 does not affect the NS1-mediated downregulation of cellular innate immune pathways in reporter gene assays, leaving in question the exact biological role and relevance of the UAP56–NS1 association.

## Introduction

UAP56, a member of the DExD/H-box RNA helicase family, is highly conserved from yeast to humans and plays a critical role in pre-mRNA splicing and mRNA nuclear export (Luo et al., 2001). The ATPase and unwinding activities of UAP56 are required for spliceosome assembly and maturation (Shen et al., 2007, 2008). In addition, UAP56 functions as a component of the transcript export (TREX) complex to efficiently export spliced mRNAs to the cytoplasm (Jensen et al., 2001; MacMorris et al., 2003). URH49, a 90% homologous paralog of UAP56, has similar functions in mRNA processing (Pryor et al., 2004; Kapadia et al., 2006).

Influenza viruses replicate in the nucleus of infected cells and usurp the cellular nuclear export systems for the transport of viral mRNA from the nucleus to the cytoplasm. Accordingly, they rely on host factors such as UAP56/URH49 for efficient replication (Read and Digard, 2010; Wisskirchen et al., 2011b). UAP56/URH49 interacts with the influenza viral nucleoprotein (NP) to facilitate the formation of viral ribonucleoprotein complexes (Momose et al., 2001; Kawaguchi et al., 2011), and interacts with the cellular MxA protein (Wisskirchen et al., 2011a), an interferon-induced dynamin-like GTPase that restricts the replication of influenza A viruses (Pavlovic et al., 1992; Haller et al., 2015) by directly interacting with the viral NP protein (Manz et al., 2013). However, the significance of the UAP56/URH49–MxA interaction for the antiviral effect of MxA is unknown. UAP56/URH49-depletion has been shown to increase the accumulation of double-stranded RNA (dsRNA) in influenza virus-infected cells, resulting in the activation of protein kinase RNA (PKR; Wisskirchen et al., 2011b), a known antiviral factor (Gale and Katze, 1998). UAP56/URH49 may, therefore, be involved in regulating host antiviral responses in influenza virus-infected cells.

NS1 performs several roles in the influenza A viral life cycle (Wright et al., 2013). It is involved in the nuclear export of cellular and viral mRNAs (Fortes et al., 1994; Qiu and Krug, 1994) and is the major influenza A viral interferon (IFN) antagonist (Garcia-Sastre et al., 1998; Krug, 2015). It downregulates host antiviral responses by suppressing the activation of viral RNA receptor retinoic acid-inducible gene I (RIG-I; Pichlmair et al., 2006; Mibayashi et al., 2007; Opitz et al., 2007; Gack et al., 2009) and the downstream adaptor-protein IFN-beta promoter stimulator protein 1 (IPS-1; Mibayashi et al., 2007), resulting in reduced activation of IFN regulatory factor 3 (IRF3), a critical transcription factor for type-I IFN gene induction (Talon et al., 2000). Furthermore, NS1 inhibits PKR (Lu et al., 1995) and 2′-5′-oligoadenylate synthetase (OAS; Min and Krug, 2006), both of which are IFN-induced antiviral effector proteins. NS1 also affects viral replication through its PDZ domain binding motif (Jackson et al., 2008; Soubies et al., 2010) and by activating the PI3K/Akt pathway (Ehrhardt et al., 2006, 2007; Hale et al., 2006; Shin et al., 2007a,b,c). Moreover, NS1 binding to the 30-kDa subunit of the cellular cleavage and polyadenylation specificity factor CPSF30 leads to the nuclear accumulation of cellular mRNAs (including mRNAs encoding IFN) and hence to lower levels of these proteins (Nemeroff et al., 1998; Noah et al., 2003). Since NS1 and UAP56 affect similar cellular processes including pre-mRNA splicing, mRNA nuclear export, and the indirect suppression of antiviral responses, we asked whether UAP56 associates with NS1 and/or affects functions of NS1.

## Materials and Methods

### Cells, Viruses, and Plasmids

Human embryonic kidney HEK293T cells were maintained in DMEM supplemented with 10% of fetal calf serum (FCS). Adenocarcinomic human alveolar basal epithelial (A549) cells were maintained in DMEM/F-12 supplemented with 10% FCS. Madin–Darby canine kidney (MDCK) cells were maintained in Eagle’s minimal essential medium (MEM) supplement with 5% newborn calf serum (NCS). Influenza A/WSN/1933 virus (WSN; H1N1) and WSN-NS1-R38A-K41A mutant virus were generated by using reverse genetics (Neumann et al., 1999) and propagated in MDCK cells. The protein coding regions of human UAP56 and MxA were cloned into the pCAGGS protein expression vector (Niwa et al., 1991) with or without an in-frame FLAG-tag at the 5′-end. UAP56 mutants K95A, K95N (which lack ATPase activity due to defective ATP-binding; Kota et al., 2008; Shen et al., 2008), E197A (which lacks ATPase activity due to defective ATP hydrolysis), and D199A (with increased ATPase activity; Shen et al., 2008) were generated by using the PrimeSTAR Mutagenesis Basal Kit (Takara Bio). The protein expression plasmid encoding WSN-NS1 protein was described previously (Watanabe et al., 2014). The coding regions of the NS1 proteins from influenza A/Brevig Mission/1/1918 (H1N1; BM/1/1918), A/Vietnam/1203/2004 (H5N1; VN1203), and A/Anhui/1/2013 (H7N9; AH/1) were cloned into the pCAGGS vector. WSN-NS1 mutants R38A-K41A, F103L-M106I, and ΔPDM (deletion of the PDZ domain-binding motif, PDM, amino acids 227–230 of WSN NS1) were generated by using the PrimeSTAR Mutagenesis Basal Kit (Takara Bio). All viruses and plasmids were sequenced prior to their use to confirm the identity of the desired sequences. No animals, or human or animal samples were used in this study.

### Co-immunoprecipitation

HEK293T cells were transfected with the indicated pCAGGS vectors and TransIT-LT1 (Mirus). At 48 h post-transfection, the cells were lyzed in lysis buffer [20 mM Tris-HCl (pH7.5), 100 mM NaCl, 0.5% Triton X-100, and protease inhibitor cocktail (Sigma)] on ice for 10 min. After centrifugation at 15,300 *g* for 10 min at 4°C, the supernatant was collected. Co-immunoprecipitation was performed by incubation with antibody (4°C, overnight) and subsequently with Dynabeads Protein G (Life Technologies) for 20 min, or by incubation with anti-FLAG M2 magnetic beads (Sigma) at 4°C overnight. Rabbit anti-FLAG antibody (F7425; Sigma), or mouse anti-FLAG M2 antibody (F1804; Sigma) were used for the immunoprecipitation. Dynabeads Protein G beads were suspended in 6x Laemmli buffer [375 mM Tris-HCl, 9% SDS, 50% glycerol, 0.03% bromophenol blue, 6% 2-mercaptoethanol] and heated at 98°C for 5 min. Anti-FLAG M2 magnetic beads were incubated in buffer [20 mM Tris-HCl (pH7.5), 100 mM NaCl] containing 100 μg/mL FLAG peptide (Sigma) and the supernatant was suspended in Laemmli buffer and heated at 98°C for 5 min.

### Immunoblotting

Proteins extracted from the cells were separated by SDS-PAGE and transferred onto PVDF membranes (Invitrogen). Anti-UAP56 antibody (ab1811061; abcam), anti-FLAG M2 antibody (F1804; Sigma), anti-β-actin antibody (A5316; Sigma), and anti-Mx1 antibody (ab95926; abcam) were used for immunoblotting. Wild-type (WT) and mutant WSN-NS1 proteins were analyzed with anti-influenza A NS1 antibody sc-130568 (Santa Cruz) and/or GTX125990 (GeneTex). Blots were developed using Lumi-Light Western blotting substrate (Sigma) or SuperSignal West Femto Maximum sensitivity substrate (Thermo), and exposed to X-ray film Super RX-N (FUJI film) or analyzed by AlphaImager (Alpha Innotech).

### RNase Susceptibility Assay

HEK293T cells were transfected with pCAGGS vectors encoding WSN-NS1 and FLAG-tagged UAP (pCAGGS-FLAG-UAP56) or with the pCAGGS control vector. At 48 h post-transfection, cells were lyzed in lysis buffer containing 2 mM MgCl_2_ and cell lysate was treated without or with 40 U/mL RNase III (BioLabs), 200 μg/mL RNase A (Thermo Scientific), or 40 U/mL RNase H (Invitrogen) at 37°C for 20 min. The lysates were then incubated with anti-FLAG antibody-conjugated magnetic beads (4°C, overnight), and co-precipitated proteins were analyzed by immunoblotting.

### Indirect Immunofluorescent Analysis

A549 cells were infected with WT WSN or WSN-NS1-R38A-K41A mutant virus at a multiplicity of infection (MOI) of three. At the indicated time points post-infection, cells were fixed with 4% paraformaldehyde in PBS and permeabilized with 0.1% Triton X-100 in PBS. Mouse anti-UAP56 antibody (LS-C172345; LSBio) and rabbit anti-NS1 antibody (PA5-32243; Thermo Fisher) were used as primary antibodies. Alexa 488-conjugated anti-rabbit and Alexa 594-conjugated anti-mouse antibodies (Life Technologies) were used as secondary antibodies. Slides were mounted in mounting media with DAPI, and analyzed by using LSM510 META (Carl Zeiss). The immunofluorescence co-localization of NS1 and UAP56 was assessed by Pearson’s correlation coefficient of red- and green-pixels, calculated by using the FIJI “coloc2” function^1^ (Schindelin et al., 2012).

### Growth Kinetics of Viruses in Cell Culture

African green monkey kidney (Vero) cells, and hepatocarcinoma cell lines Huh7.0 and Huh7.5 (a derivative of Huh7.0 cells bearing a defective form of RIG-I; Sumpter et al., 2005) were transfected with siRNA targeting UAP56 (Hs_BAT1_5 FlexiTube siRNA; QIAGEN) or control siRNA (AllStars Negative Control siRNA; QIAGEN) with Lipofectamine RNAiMAX reagent. At 24 (Vero cells) or 48 h (Huh7.0 and Huh7.5 cells) post-transfection, the cells were infected with WSN virus or WSN-NS1-R38A-K41A mutant virus at an MOI of 0.01 for 1 h. After the infection, the cells were incubated with 0.25 μg/mL *N*-tosyl-_L_-phenylalanine chloromethyl ketone (TPCK)-trypsin. Supernatants were collected at the indicated time points and subjected to virus titration by use of plaque assays in MDCK cells.

### Reporter Assays to Assess the Inhibitory Effect of NS1 on Innate Immune Responses

To assess NS1’s ability to suppress IFN-β promoter activity, HEK293T cells were transfected with p125Luc (a plasmid encoding firefly luciferase under the control of the IFN-β promoter; kindly provided by Dr. T. Fujita, Kyoto University, Japan), and with pRL-TK (a plasmid encoding Renilla luciferase under the control of the herpes simplex virus thymidine kinase promoter; Promega) as an internal transfection control. Cells were co-transfected with a constitutively active form of RIG-I (pCAGGS-RIG-I N; Yamayoshi et al., 2015), without or with increasing amounts of the protein expression plasmids for WSN-NS1 (pCAGGS-NS1) and UAP56 (pCAGGS-UAP56). The cells were cultured at 37°C, and 24 h later lyzed to measure Firefly-luc and Renilla-luc (internal control) activity by using the Dual-Glo Luciferase assay system (Promega). Firefly luciferase activity was normalized to Renilla luciferase values. The pCAGGS control vector was added as needed to ensure that all wells of cells were transfected with the same amount of DNA.

To measure NS1’s ability to interfere with the activation of IFN-stimulated response elements (ISREs), HEK293T cells were transfected with a plasmid expressing firefly luciferase under the control of an ISRE (pISRE-Luc) and with pRL-TK (internal control) with increasing amounts of protein expression plasmids for WSN-NS1 (pCAGGS-NS1) and UAP56 (pCAGGS-UAP56). At 24 h post-transfection, the cells were treated without or with IFN-β (10,000 U/mL) and cultured for 24 h. Then, the cells were lyzed and the luciferase activity of the Firefly-luc and internal control Renilla-luc were analyzed as described above.

## Results

### UAP56 Associates With NS1

**FIGURE 1:**
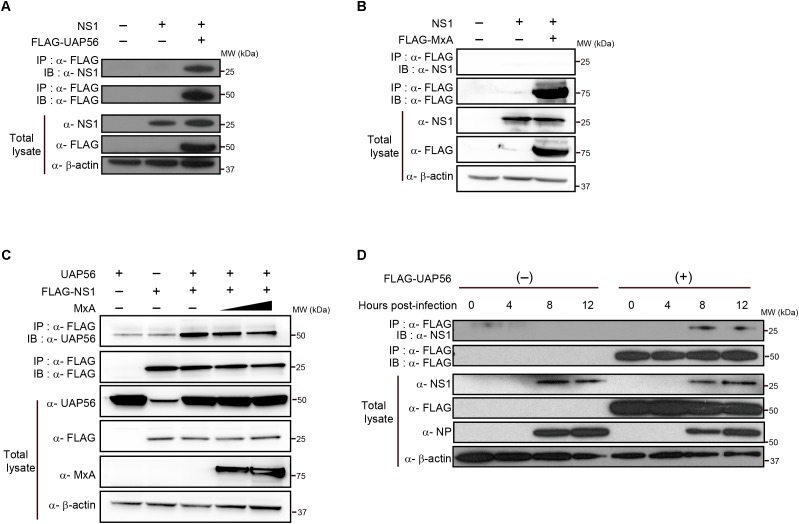
*In vitro* coprecipitation of NS1 with UAP56 or MxA. **(A)** *In vitro* interaction of NS1 with UAP56. HEK293T cells were transfected with protein expression plasmids for WSN-NS1 and/or FLAG-tagged UAP56. At 48 h post-transfection, cells were lyzed and immunoprecipitated (IP) with anti-FLAG antibody. Co-precipitated proteins were analyzed by immunoblotting (IB) with anti-NS1 antibody. **(B)** *In vitro* interaction of NS1 with MxA. HEK293T cells were transfected with protein expression plasmids for WSN-NS1 and/or FLAG-tagged MxA. Co-immunoprecipitation and IB were carried out as described in **A**. **(C)** Overexpression of MxA does not affect the UAP56–NS1 interaction. HEK293T cells were transfected with plasmids expressing UAP56 and/or FLAG-tagged NS1, and increasing amounts of MxA. Forty-eight hours later, the cells were lyzed and IP with anti-FLAG antibody. Co-immunoprecipitation and IB were carried out as described in **A**. **(D)** UAP56–NS1 interaction in influenza A virus-infected cells. HEK293T cells were transfected with pCAGGS-FLAG-UAP56 or a control vector, and infected with WSN virus at a multiplicity of infection (MOI) of one. The cells were harvested and lyzed at the indicated time points post-infection and IP with anti-FLAG antibody. Co-precipitated proteins were analyzed by IB with anti-NS1 antibody; the NP expression levels in the total cell lysate were analyzed as an infection control.

First, we tested whether UAP56 associates with NS1. Human embryonic kidney HEK293T cells were transfected with protein expression vectors encoding FLAG-tagged UAP56 and the NS1 protein of A/WSN/33 (H1N1; WSN) virus. Overexpressed WSN-NS1 co-precipitated with UAP56 (**Figure 1A**), indicating an association between these two proteins. We next tested whether the UAP56–NS1 co-precipitation was affected by the overexpression of MxA. NS1 did not directly interact with MxA (**Figure 1B**), and increasing amounts of MxA did not abrogate the UAP56–NS1 co-precipitation (**Figure 1C**).

We further examined the UAP56–NS1 association in virus-infected cells. FLAG-tagged UAP56 or a control vector was transfected into HEK293T cells, followed by infection with WSN virus at a MOI of one. At different time points after infection, proteins co-precipitated with UAP56 were analyzed for NS1 (**Figure 1D**). As expected, NS1 co-precipitated with UAP56, demonstrating that NS1 associates with UAP56 in WSN virus-infected cells.

### UAP56 Associates With NS1 Proteins of Different Influenza Viruses

**FIGURE 2:**
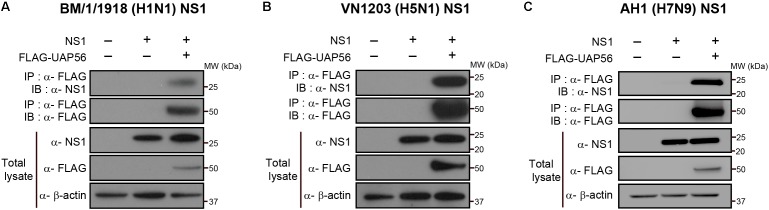
UAP56 interacts with NS1 proteins derived from different influenza A viruses. HEK293T cells were transfected with plasmids expressing FLAG-tagged UAP56 proteins and/or the NS1 proteins of A/Brevig Mission/1/1918 (H1N1; BM/1/1918) **(A)**, A/Vietnam/1203/2004 (H5N1; VN1203) **(B)**, or A/Anhui/1/2013 (H7N9; AH1) **(C)**. At 48 h post-transfection, the cells were lyzed and immunoprecipitated with anti-FLAG M2 antibody-conjugated magnetic beads. Co-precipitated proteins were analyzed by immunoblotting with anti-NS1 antibody.

To determine whether UAP56 associates with NS1 proteins from different influenza A viruses, we tested UAP56 co-precipitation with the NS1 proteins of pandemic 1918 virus (A/Brevig Mission/1/1918, H1N1; BM/1/1918), a representative of highly pathogenic avian H5N1 influenza viruses (A/Vietnam/1203/2004, VN1203), and a representative of the recently emerged avian H7N9 viruses (A/Anhui/1/2013, AH1). The NS1 proteins of all three viruses co-precipitated with UAP56 (**Figures 2A–C**), demonstrating that the UAP56 association with NS1 is not specific to WSN virus.

### The RNA-Binding Residues in NS1 Are Critical for Its Association With UAP56

**FIGURE 3:**
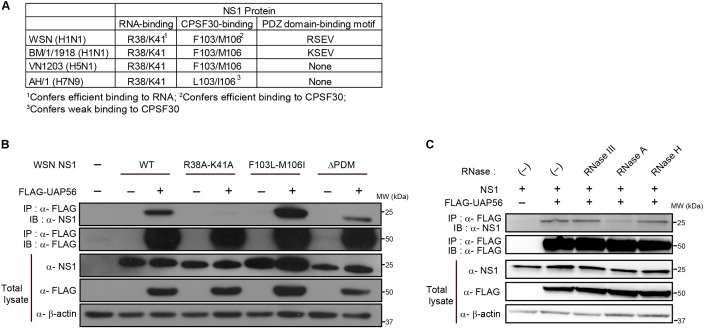
Identification of NS1 residues critical for the UAP56–NS1 interaction. **(A)** Comparison of RNA-binding residues, CPSF30-interacting residues, and PDZ domain binding motifs among the NS1 proteins of WSN, BM/1/1918, VN1203, and AH1. **(B)** Interaction of UAP with mutant NS1 proteins. HEK293T cells were transfected with a protein expression vector for wild-type (WT) or mutant WSN-NS1 protein and FLAG-tagged UAP56 or a control vector. At 48 h post-transfection, the cells were lyzed and immunoprecipitated with anti-FLAG M2 antibody-conjugated magnetic beads. Co-precipitated proteins were analyzed by immunoblotting with anti-NS1 antibody. ΔPDM: deletion of the “PDZ domain-binding motif.” **(C)** Effect of RNase treatment on the UAP56–NS1 interaction. HEK293T cells were transfected with plasmids for the expression of WSN-NS1 and FLAG-tagged UAP56. At 48 h post-transfection, the cells were lyzed and the collected cell lysate was mock-treated or treated with the indicated RNases at 37°C for 20 min. The lysates were incubated with anti-FLAG antibody-conjugated magnetic beads, and co-precipitated proteins were analyzed by immunoblotting.

To examine the amino acid residues that mediate NS1–UAP56 association, we next tested WSN-NS1 mutants encoding R38A-K41A (defective binding to RNA), F103L-M106I (defective binding to CPSF30), or lacking the PDZ domain-binding motif (ΔPDM) for their binding affinity for UAP56 in a co-immunoprecipitation assay (**Figure 3A**). Co-precipitation of UAP56 with the NS1-R38A-K41A mutant was severely reduced (**Figure 3B**), demonstrating that the RNA-binding residues in NS1 are critical for NS1-UAP56 association. By contrast, mutation of the CPSF30-binding residues or deletion of the PDZ domain binding motif did not abrogate the WSN–NS1 association with UAP56, although deletion of the PDZ domain binding motif reduced NS1’s association affinity for UAP56 relative to that of WT NS1 (**Figure 3B**).

To test the possibility that UAP56–NS1 association is mediated by nucleic acids, we examined the UAP56–NS1 association in cell lysates treated with RNase III (specific for dsRNA), RNase A (specific for ssRNA), and RNase H (specific for DNA–RNA duplexes). Treatment with RNase III or RNase H did not appreciably affect the UAP56–NS1 interaction (**Figure 3C**). By contrast, RNase A treatment significantly reduced the extent of the UAP56–NS1 association (**Figure 3C**), establishing that this association is mediated by ssRNA.

### UAP56 and NS1 Co-localize Near the Nuclear Membrane

**FIGURE 4:**
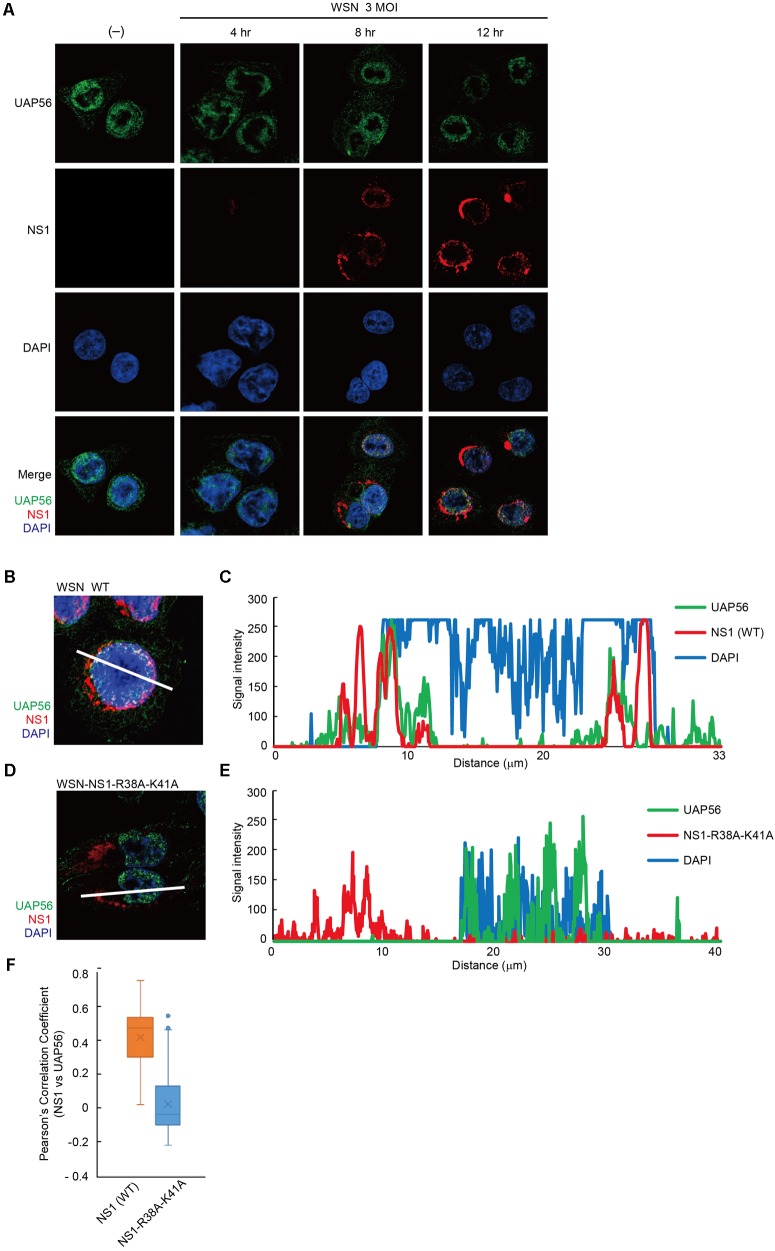
Localization of UAP56 and NS1 in WSN-infected A549 cells. **(A)** A549 cells were infected with WSN virus at an MOI of three. At 4, 8, or 12 h post-infection, the cells were fixed with 4% paraformaldehyde in PBS. The cells were then analyzed with specific antibodies against UAP56 and NS1. **(B–E)** Representative immunofluorescence images of A549 cells at 12 h post-infection with wild-type (WT) WSN virus **(B)** or WSN-NS1-R38A-K41A mutant virus **(D)** at an MOI of 3. The cells were analyzed with anti-UAP56 (green) antibody, anti-NS1 (red) antibody, and DAPI (blue). **(C,E)** Signal intensities across the line shown in **B**,**D**, respectively, were plotted and analyzed by using LSM510 META and ZEN2009 software. **(F)** A549 cells infected with WSN WT virus (*n* = 102 cells) or WSN-NS1-R38A-K41A mutant virus (*n* = 100 cells) at an MOI of three for 12 h were imaged, and Pearson’s correlation coefficients for UAP56 and NS1 co-localization were determined for individual cells.

To analyze the intracellular localization of UAP56 and NS1, we next infected human A549 cells with WSN virus and performed an indirect immunofluorescence analysis with antibodies to NS1 and UAP56. In non-infected cells, UAP56 was detected predominantly in the nucleus but was also present in the cytoplasm (**Figure 4A**, left panel). In WSN virus-infected cells, NS1 was expressed in the nucleus and cytoplasm (**Figure 4A**). Further analysis showed that WT NS1 co-localized with UAP56 near the nuclear membrane (**Figures 4B,C**). On the other hand, in cells infected with WSN-NS1-R38A-K41A mutant virus, which encodes RNA-binding defective NS1, the mutant NS1 was dominantly expressed in the cytoplasm with limited co-localization with UAP56 (**Figures 4D,E**). Quantitative co-localization analysis confirmed NS1-UAP56 association in WT WSN-infected cells, whereas this co-localization was abrogated in cells infected with WSN-NS1-R38A-K41A mutant virus (**Figure 4F**).

### UAP56 Does Not Interfere With NS1-Mediated Suppression of RIG-I-Dependent IFN-β Induction and ISG Stimulation

**FIGURE 5:**
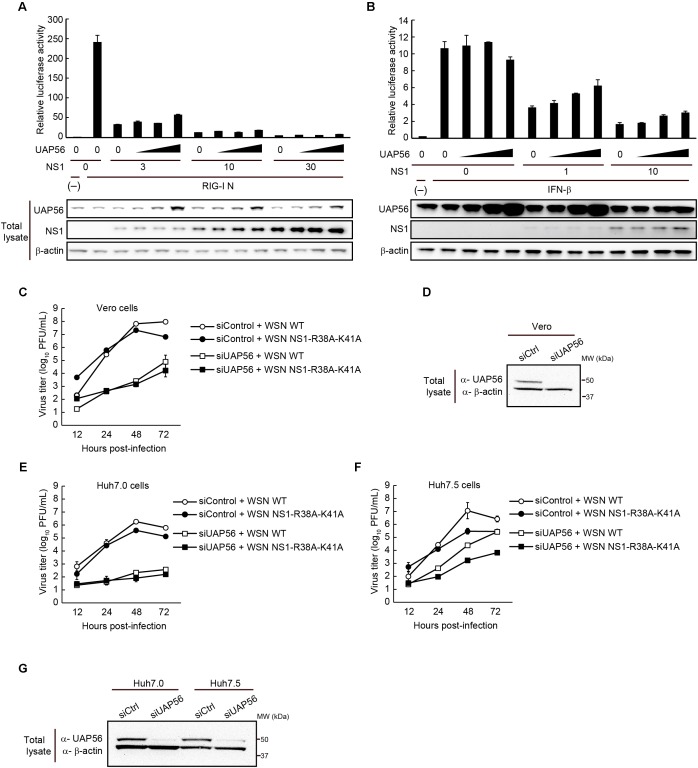
Effect of UAP56 overexpression on the IFN-antagonist activity of NS1. **(A)** UAP56 overexpression does not affect NS1’s ability to suppress IFN-β promoter activity. HEK293T cells were transfected with plasmids expressing firefly luciferase under the control of an IFN-β promoter, pRL-TK luciferase (as an internal control), a constitutively active form of RIG-I (pCAGGS-RIG-I N), increasing amounts of pCAGGS-NS1 (0, 3, 10, or 30 ng), and pCAGGS-UAP56 (0, 3, 10, or 30 ng). Twenty-four hours later, the cells were lyzed and firefly and Renilla luciferase activities were measured. The firefly luciferase activity was normalized by the internal control value. The total amount of transfected vector was adjusted in all wells using the pCAGGS control vector. Data are shown as the mean ± SD (*n* = 3; biological replicates). UAP56 and NS1 expression levels in cells were examined by immunoblotting. **(B)** UAP56 overexpression does not affect NS1’s ability to suppress ISRE-driven gene expression. HEK293T cells were transfected with plasmids expressing firefly luciferase under the control of an ISRE promoter element, pRL-TK luciferase (as an internal control), and increasing amounts of pCAGGS-NS1 (0, 1, or 10 ng) and pCAGGS-UAP56 (0, 3, 10, or 30 ng). Twenty-four hours later, the cells were mock-treated or treated with IFN-β (10,000 U/mL), and cultured for 24 h. Luciferase activities were measured and data were analyzed as described in **A**. Data are shown as the mean ± SD (*n* = 3; biological replicates). UAP56 and NS1 expression levels in cells were examined by immunoblotting. **(C–G)** WSN virus or WSN-NS1-R38A-K41A mutant virus replication in cells transfected with siRNA targeting UAP56 or with a control siRNA. Vero **(C)**, Huh7.0 **(E)**, or Huh7.5 **(F)** cells were transfected with siRNA targeting UAP56, or a control siRNA. At 24 (Vero cells) or 48 h (Huh7.0 and Huh7.5 cells) post-transfection, the cells were infected with WSN virus or WSN-NS1-R38A-K41A mutant virus at an MOI of 0.01 and incubated at 37°C. Culture supernatants were collected at the indicated times post-infection and viral titers were analyzed by performing plaque assays in MDCK cells. The data are shown as the mean ± SD (*n* = 3; biological replicates). Levels of UAP56 expression in Vero **(D)** or Huh7.0 and Huh7.5 **(G)** cells transfected with siRNA targeting UAP56, or with a control siRNA. UAP56 expression levels were analyzed by immunoblotting at 48 (Vero cells) or 72 h (Huh7.0 and Huh7.5 cells) after siRNA transfection.

To test whether overexpression of UAP56 affects the immune suppressive functions of NS1, we transfected HEK293T cells with a reporter plasmid expressing firefly luciferase under the control of an IFN-β promoter. These cells were also transfected with a vector expressing a constitutively active form of RIG-I, and with vectors expressing increasing amounts of NS1 and UAP56 (**Figure 5A**). WSN NS1 suppressed RIG-I-dependent IFN-β promoter activation, but this activity was not significantly affected by increasing amounts of co-expressed UAP56.

Next, we tested the effect of increasing amounts of UAP56 on NS1’s ability to suppress the induction of ISGs. Cells were transfected with a reporter plasmid possessing the firefly luciferase gene under the control of a promoter with an ISRE, and with increasing amounts of plasmids expressing NS1 or UAP56. Twenty-four hours later, the cells were stimulated with IFN-β for 24 h, and then assayed for luciferase expression. NS1 expression suppressed the activation of IFN-β-activated genes, but co-expression of UAP56 did not appreciably alter this effect (**Figure 5B**).

Finally, we assessed influenza virus titers in Vero cells, which are defective in IFN-α/β secretion. As shown in **Figures 5C,D**, UAP56 downregulation also restricted WSN virus growth in Vero cells. WSN-NS1-R38A-K41A mutant virus, which encodes RNA-binding defective NS1, was also restricted by UAP56 downregulation. We also examined the growth of these viruses in the hepatocarcinoma cell lines Huh7.0 and Huh7.5 (a derivative of Huh7.0 cells which carry a defective form of RIG-I). As shown in **Figures 5E–G**, in both cell lines, WSN virus growth was restricted by UAP56 down-regulation.

## Discussion

UAP56/URH49 facilitates the efficient replication of influenza A viruses through its roles in pre-mRNA splicing, mRNA nuclear export, and the indirect suppression of cellular antiviral responses (Luo et al., 2001); these functions are also affected by the viral NS1 protein (Fortes et al., 1994; Qiu and Krug, 1994). Here, we demonstrated that the cellular protein UAP56 associates with the influenza viral protein NS1. NS1 also co-precipitated with FLAG-tagged URH49 (data not shown), suggesting that UAP56 and URH49 exhibit redundancy. Because we were unable to obtain anti-URH49 antibodies of high quality, we focused on UAP56 in this study. Since UAP56 interacts with MxA (Wisskirchen et al., 2011a), which suppresses influenza A virus replication (Pavlovic et al., 1992; Haller et al., 2015), we tested the possibility that UAP56–NS1 association is disturbed by MxA. NS1–MxA co-precipitation was not detected, and MxA overexpression did not affect the UAP56–NS1 co-precipitation. These results demonstrate that UAP56–NS1 association is not affected by MxA.

UAP56 was co-precipitated with the NS1 proteins of different influenza viruses including the pandemic 1918 virus, a representative of highly pathogenic H5N1 viruses which are now enzootic in poultry populations in Southeast Asia and have to date caused at least 860 human infections^2^, and a representative of the H7N9 viruses that are circulating in poultry in China and have caused five waves of human infections^3^ to date. These results demonstrated that UAP56 association with NS1 is not specific to WSN virus.

Several amino acid residues in NS1 have been shown to interact with host proteins and/or affect the biological functions of NS1 (Marc, 2014). Among them are the RNA-binding residues R38 and K41 (Wang et al., 1999), the CPSF30-binding residues 103 and 106, and the PDZ domain binding motif (located at the C-terminus of most influenza A virus NS1 proteins) (Jackson et al., 2008; Liu et al., 2010; Zielecki et al., 2010; Fan et al., 2013). The PDZ domain binding motif is not present in the VN1203 and AH1 NS1 proteins, whereas the WSN and BM/1/1918 NS1 proteins encode PDZ domain binding motifs with the sequences RSEV and KSEV, respectively; the latter contributes to the virulence of the BM/1/1918 virus (Jackson et al., 2008). We tested UAP56 co-precipitation with WSN-NS1 mutants encoding R38A-K41A (defective binding to RNA; Wang et al., 1999), F103L-M106I (defective binding to CPSF30; Kochs et al., 2007; Ping et al., 2011), or lacking the PDZ domain binding motif (ΔPDM). Co-precipitation of the NS1-R38A-K41A mutant with UAP56 was severely reduced, demonstrating that the RNA-binding residues in NS1 are critical for NS1-UAP56 association. Arginine at position 38 and a basic amino acid at position 41 are highly conserved among influenza A virus NS1 proteins, likely explaining why the UAP56–NS1 association is conserved among the NS1 proteins of different virus origins.

The NS1 amino acids R38 and K41 interact with several cellular and viral RNA species, such as small nuclear RNA, dsRNA, single-stranded RNA (ssRNA), viral RNA, and DNA–RNA duplexes (Hatada and Fukuda, 1992; Lu et al., 1994; Wang and Krug, 1998; Saguez et al., 2013; Anastasina et al., 2016). Treatment with RNase A (specific for ssRNA) abrogated the UAP56–NS1 association, suggesting that RNA-bound NS1 associates with UAP56 or that the UAP56–NS1 association is indirectly mediated by ssRNA.

In an indirect immunofluorescence analysis, UAP56 was predominantly localized in the nucleus but was also present in the cytoplasm in non-infected cells, consistent with previous studies (Kota et al., 2008; Wisskirchen et al., 2011a). WT NS1 co-localized with UAP56 near the nuclear membrane in WT-WSN virus-infected cells, whereas mutant NS1 was predominantly expressed in the cytoplasm with limited co-localization with UAP56 in WSN-NS1-R38A-K41A mutant virus. Quantitative co-localization analysis showed that NS1–UAP56 association in WT WSN-infected cells was abrogated in cells infected with WSN-NS1-R38A-K41A mutant virus, consistent with our in vitro co-immunoprecipitation analysis.

Among its many functions, NS1 suppresses RIG-I-dependent IFN-β induction and the activation of ISGs (Egorov et al., 1998; Garcia-Sastre et al., 1998; Rajsbaum et al., 2012), therefore we tested the effect of UAP56 overexpression on the immune suppressive functions of NS1 in a reporter gene assay. WSN NS1 suppressed RIG-I-dependent IFN-β promoter activation, but this activity was not significantly affected by increasing amounts of co-expressed UAP56. Also, in a reporter gene assay under the control of a promoter with an ISRE, NS1 expression suppressed the activation of IFN-β-activated genes, which was not significantly altered by co-expression of UAP56. Together, these data indicate that UAP56 does not significantly affect the immune suppressive functions of NS1.

Wisskirchen et al. (2011b) demonstrated that siRNA-mediated downregulation of UAP56 reduced influenza virus titers in IFN-competent cells. UAP56 downregulation also restricted WSN virus growth in Vero cells, which are defective in IFN-α/β secretion, suggesting that the supportive function of UAP56 in influenza A virus replication does not require a functional IFN system. WSN-NS1-R38A-K41A mutant virus was also restricted by UAP56 downregulation, further suggesting that the UAP56–NS1 association was not related to UAP56’s essential role in influenza virus replication. We also examined the growth of these viruses in a cell line that carries a defective form of RIG-I. WSN virus growth was also restricted by UAP56 down-regulation in this cell line, indicating that UAP56’s supportive function in influenza A virus replication is not mediated by NS1’s suppressive effect on RIG-I. The limited replication of WSN-NS1-R38A-K41A upon UAP56 downregulation further suggests that the UAP56–NS1 association is not essential for UAP56 function.

The UAP56–NS1 association may be related to the regulation of mRNA splicing and/or nuclear export, consistent with the known roles of NS1 (Fortes et al., 1994; Qiu and Krug, 1994; Satterly et al., 2007) and UAP56 (Jensen et al., 2001; Herold et al., 2003; MacMorris et al., 2003). This assumption is further supported by the co-localization of these two proteins near the nuclear membrane (**Figures 4B,C**), while NS1 counteracts the host immune responses mainly in cytoplasm. It is also supported by our finding that the RNA-binding residues of NS1 (i.e., NS1-R38/K41) are required for the UAP56–NS1 association. However, a functional relationship between UAP56 and NS1 has yet to be shown.

It was previously reported that influenza B virus NS1 colocalizes with nuclear mRNA splicing speckles in the nucleus, and that this localization pattern is different from that of influenza A NS1 (Schneider et al., 2009). We found that the ATP-binding/-hydrolyzing residues in UAP56, which are essential for UAP56 co-localization with splicing complexes, are not required for the co-precipitation with influenza A virus NS1 (**Supplementary Figure S1**). These results suggest that the association of UAP56 with influenza A NS1 may be functionally different from that of UAP56 with influenza B virus NS1. The biological importance and exact role of the UAP56–NS1 association in the life cycle of influenza A viruses remain to be assessed.

## Author Contributions

SC and LH-B performed the experiments. SC, LH-B, GN, and YK planned the experiments and analyzed the data. SC, GN, and YK wrote the manuscript.

## Conflict of Interest Statement

The authors declare that the research was conducted in the absence of any commercial or financial relationships that could be construed as a potential conflict of interest.

## Footnotes

1 https://fiji.sc/

2 http://www.who.int/influenza/human_animal_interface/Influenza_Summary_IRA_HA_interface_02_03_2018.pdf?ua=1

3 http://www.fao.org/ag/againfo/programmes/en/empres/h7n9/situation_update.html

## Supplementary Material

FIGURE S1 *In vitro* co-precipitation of NS1 with UAP56 mutants. HEK293T cells were transfected with a protein expression vector for wild-type (WT) WSN-NS1 protein and FLAG-tagged WT or mutant UAP56 protein or a control vector. K95A and K95N: UAP56 mutants which lack ATPase activity due to defective ATP-binding; E197A: UAP56 mutant which lacks ATPase activity due to defective ATP hydrolysis; D199A: UAP56 mutant with increased ATPase activity. At 48 h post-transfection, the cells were lysed and immunoprecipitated with anti-FLAG M2 antibody-conjugated magnetic beads. Co-precipitated proteins were analyzed by immunoblotting with anti-NS1 antibody.
