# Supplementary figures and images for: The Cellular DExD/H-Box RNA Helicase UAP56 Co-localizes With the Influenza A Virus NS1 Protein
Source: Front Microbiol. 2018 Sep 12;9:2192. doi: 10.3389/fmicb.2018.02192 (PMC6144874; doi:10.3389/fmicb.2018.02192)

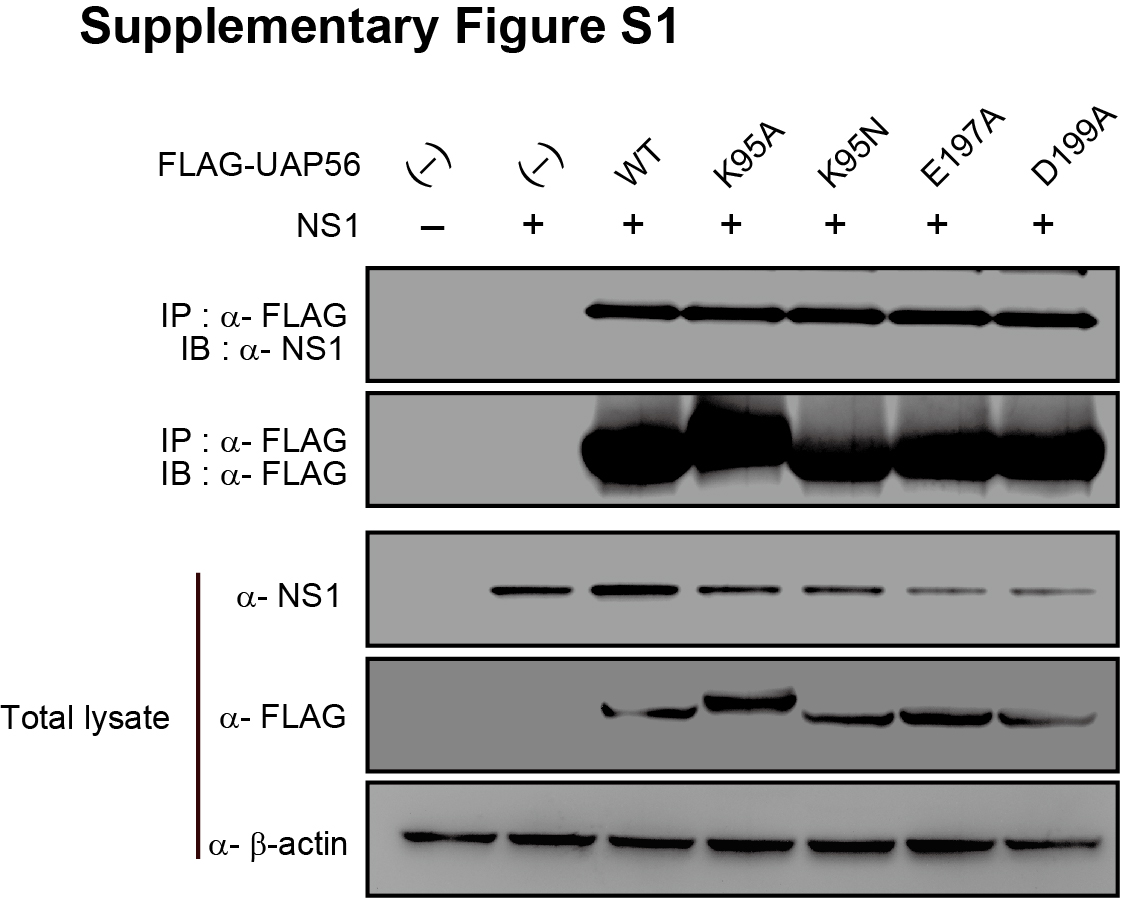

Supplement: FIGURE S1 — In vitro co-precipitation of NS1 with UAP56 mutants. HEK293T cells were transfected with a protein expression vector for wild-type (WT) WSN-NS1 protein and FLAG-tagged WT or mutant UAP56 protein or a control vector. K95A and K95N: UAP56 mutants which lack ATPase activity due to defective ATP-binding; E197A: UAP56 mutant which lacks ATPase activity due to defective ATP hydrolysis; D199A: UAP56 mutant with increased ATPase activity. At 48 h post-transfection, the cells were lysed and immunoprecipitated with anti-FLAG M2 antibody-conjugated magnetic beads. Co-precipitated proteins were analyzed by immunoblotting with anti-NS1 antibody. [file Image_1.JPEG]
